# Timing of intermittent preventive treatment for malaria during pregnancy and the implications of current policy on early uptake in north-east Tanzania

**DOI:** 10.1186/1475-2875-7-79

**Published:** 2008-05-09

**Authors:** Katherine Anders, Tanya Marchant, Pili Chambo, Pasiens Mapunda, Hugh Reyburn

**Affiliations:** 1 London School of Hygiene and Tropical Medicine, London, WC1E 7HT, UK; 2 Joint Malaria Programme, PO Box 2228, KCMC, Moshi, Tanzania; 3 Centre for Enhancement of Effective Malaria Interventions, National Institute for Medical Research, P.O. Box 9653, Dar Es Salaam, Tanzania

## Abstract

**Background:**

Intermittent preventive treatment (IPTp) is efficacious in reducing the adverse outcomes associated with pregnancy-associated malaria, however uptake of the recommended two doses is low in Tanzania, and little is known of the timepoint during pregnancy at which it is delivered. This study investigated the timing of delivery of IPTp to pregnant women attending antenatal clinics (ANC), and the potential determinants of timely uptake.

**Methods:**

Structured interviews were conducted with staff and pregnant women at antenatal clinics in northeast Tanzania, and antenatal consultations were observed. Facility-based and individual factors were analysed for any correlation with timing of IPTp uptake.

**Results:**

Almost half the women interviewed first attended ANC during or before the fourth month of gestation, however 86% of these early attendees did not receive IPTp on their first visit. The timing of IPTp delivery complied closely with the national guidelines which stipulate giving the first dose at 20–24 weeks gestation. Uptake of at least one dose of IPTp among women who had reached this gestation age was 67%, although this varied considerably between clinics. At one facility, IPTp was not delivered because SP was out of stock.

**Conclusion:**

Early uptake of IPTp was found to be hampered by factors external to health worker performance or women's individual preferences. These include insufficient drug stocks and an apparent lack of information to health workers on the reasoning for continued use of SP for IPTp when it has been replaced as a first-line treatment. In addition, an unexpectedly high proportion of women attend antenatal clinics before 20 weeks of pregnancy. While current policy denies the use of IPTp at this time, there is emerging, but incomplete, evidence that malaria in early pregnancy may contribute considerably to the burden of pregnancy-related malaria. Current policy may thus result in a missed opportunity for maximising the benefit of this intervention, and efforts to encourage earlier attendance at ANC alone are unlikely to improve uptake of IPTp. More evidence is needed to weigh the benefits of early IPTp use against theoretical risks of antifolate drugs in early pregnancy.

## Background

Malaria infection during pregnancy has adverse consequences for both the woman and foetus, accounting for an estimated 26% of severe maternal anaemia in sub-Saharan Africa [1] and causing an estimated 100,000 – 200,000 infant deaths each year through low birth weight [2,3]. To address this burden, the World Health Organization recommends a package of interventions including intermittent preventive treatment (IPTp) with the anti-malarial drug sulphadoxine-pyrimethamine (SP) at least twice during the second and third trimesters of pregnancy, use of insecticide-treated bednets (ITNs) and effective case management [4]. The efficacy of IPTp in reducing maternal parasitaemia and anaemia has been demonstrated in several studies[5-7], and the WHO strategy has now been adopted by almost all malaria-endemic countries in Africa [8].

In Tanzania, it is national policy to offer SP to all pregnant women attending antenatal clinics [9] at between 20 and 24 weeks gestation for the first dose and between 28 and 32 weeks for the second dose [10]. It is estimated that uptake of IPTp amongst Tanzanian women who delivered in 2005/06 was 70% for one dose but only 35% for two doses [11]. Previous studies have reported a range of factors associated with incomplete coverage of IPTp, including late first attendance at the antenatal clinic [12,13], shortages of staff or drug supply, poor health worker skills, informal charges for IPTp, and inequitable access to ANC services [12]. Individual characteristics that have been found to correlate with no or incomplete IPTp uptake are multigravidity [14] and lack of knowledge of the consequences of malaria in pregnancy [15].

Relatively little is known about the timing of delivery of the first dose of IPTp to pregnant women, however this may influence whether subsequent doses are given and may also be important in terms of time spent at risk of malaria, given the growing evidence that malaria in early pregnancy may be the cause of a significant proportion of the burden of pregnancy-related malaria. There is evidence that parasitaemia is highest in the second trimester of pregnancy [16] and that malaria infection during the first trimester may lead to low birthweight [17], and thus the efficacy of IPTp may depend not only on overall uptake, but also the time point during pregnancy at which it is received. This study examined the timing of delivery of IPTp to pregnant women attending antenatal clinics in northeast Tanzania, in the context of national, individual and facility-related determinants of timely uptake of IPTp.

## Methods

### Study design and population

Data were collected on the delivery of intermittent preventive treatment during pregnancy from three sources: exit interviews with pregnant women attending antenatal clinics, interviews with antenatal clinic staff and observations of consultations at antenatal clinics. The study was conducted over a three-week period during July 2006, at six antenatal facilities in three districts within the Tanga region of northeast Tanzania: Korogwe, Pangani and Tanga. The choice of region was based on its high malaria transmission characteristics [18] and the districts were purposively selected to represent urban, peri-urban and rural settings respectively, which may be associated with different malaria transmission patterns and/or access to health care facilities and preventive interventions. Malaria transmission in this region, measured by the mean number of infected bites per person per year (entomological inoculation rate) has been estimated at 100 to 500, although this may be reduced by up to 75% where insecticide-treated bednets are widely used [19]. Prevalence of parasitaemia in the adult population in the study area has been estimated at greater than 20% [20]. Coverage of insecticide-treated nets has been measured at 16% across the >12 year-old population in one district of Tanga region during the period 2001–2005 [21] and at 18% among pregnant women in Tanzania as a whole at the time of the current study [11].

Within each district, two health facilities providing antenatal services were selected following consultation with the respective District Medical Officer – in each case the district hospital plus one smaller facility. This was intended to be a small descriptive study that would highlight potential areas for subsequent larger surveys. Within the time and resources available it was not possible to obtain a large and random sample of facilities, which would have permitted better generalizability of the results to the broader population. However, the facilities chosen represent the range of levels and settings of health care at which antenatal services are provided, which permits at least an evaluation of the variability in practices between health facilities.

All women attending a study site antenatal clinic on the two scheduled days were approached for recruitment into the study, and written informed consent was obtained from all participants. A sample of consultations at each antenatal clinic were observed, and two health staff were interviewed at each facility. Written informed consent was obtained from all participants.

### Survey procedures

Interviews were conducted in Kiswahili. Study participants were asked a series of closed questions about their socio-economic background, pregnancy history, attendance at antenatal clinic and their use of malaria preventive interventions throughout pregnancy, as well as several open questions exploring reasons for not having used certain interventions. Interviews with health workers consisted of a mixture of closed and open questions, addressing the attitudes and practices of staff in discussing malaria with pregnant women, and administering IPTp. Notes on observed consultations were taken according to pre-determined categories of interest to the study, which were discussions on malaria in pregnancy and the use of intermittent preventive treatment to protect against malaria.

### Data analysis

Data were double entered and validated using EpiData version 3.1 (the EpiData Association, Denmark) and analysed in Stata 9.0 (StataCorp LP, USA). Qualitative data from interviews was re-coded numerically to permit quantitative analysis of the key themes, using a consistent and exhaustive coding frame developed retrospectively to reflect the full range of responses. The majority of analyses were descriptive; Chi-squared tests were used to compare frequencies of outcomes of interest between facilities and to seek evidence for an association between key potential explanatory variables and the study outcomes.

## Results

Study population

A total of 133 pregnant women were recruited to the study across the six sites, of whom 119 were interviewed (Table 1). Approximately half of the total respondents came from the two busy urban antenatal clinics (ANC) in Tanga district, as the sample size at each facility was determined by the number of women attending on the study days. Loss of some study participants between recruitment and interview occurred in all but one of the sites, and was due largely to the participant leaving the clinic directly after her consultation instead of being directed to the interview room.

**Table 1:** Characteristics of study population by facility

**District (setting)**	**Pangani (rural)**		**Tanga (urban)**		**Korogwe (peri-urban)**		**TOTAL**
	
**Facility level**	**District Hospital**	**Dispensary**	**District Hospital**	**Health Centre**	**District Hospital**	**Dispensary**	
**Women interviewed**	17	13	32	28	19	10	**119**^†^
**Primigravid**	35.3%	30.8%	34.4%	28.6%	31.6%	10.0%	**30.3%**
**Attended health education class so far this pregnancy**	52.9%	75.0%	65.6%	100%	57.9%	60.0%	**71.2%**
**Median years of age (range)**	22 (17–38)	20 (17–35)	26 (18–40)	23.5 (18–36)	25 (16–36)	27.5 (19–44)	**24 (16–44)**
**Median month of gestation (range)**	5 (3–9)	7 (3–10)	6 (3–9)	6 (3–9)	7 (2–10)	6 (4–9)	**6 (2–10)**

^†^An additional 14 women across 5 of the 6 study sites were recruited to the study but did not return for interview following their consultation

The median age of respondents was 24 years (range 16 – 44 years). The educational level of the study population varied between sites, with the proportion having completed primary education ranging from 46% to 92% (average of 76%). Approximately 30% of all respondents were in their first pregnancy, with the median month of gestation 6 months (range 2 – 10 months). Thirty-nine percent of respondents were attending the antenatal clinic for the first time on the day of interview, with a median of two ANC visits made by respondents (range 1–6). A total of 50 consultations were observed across the study sites, which represented 38% of the total participants (range 0 – 100%), and between one and three staff were interviewed at each clinic.

### Attendance at antenatal clinics

On interview 75% of respondents reported having first attended an antenatal clinic during the second trimester of pregnancy, and 93% had attended at least once before the third trimester. Among the eight respondents (7%) whose first ANC visit was later than the second trimester, the reasons given for late attendance were: having had no problems during pregnancy and therefore no need to visit the clinic; a long distance to travel from home to the clinic; inability to leave farm work to travel into town; and thinking she was earlier on in gestation than she actually was.

To further investigate the timeliness of ANC attendance, a categorization of 'early first attendance' was defined as a first visit to ANC at or before 4 months gestation, compared to the national average of 19.6 weeks [11] and prior to the scheduled delivery of the first IPTp dose of 20–24 weeks [10]. Across all six facilities, almost half (48%) of all respondents had attended ANC at or before four months of gestation, within a range of 40% to 70% at individual facilities. Primigravid respondents were more likely than multigravid women to have attended ANC early (63.9% vs 41.5%; chi-squared test P = 0.025), but no other determinants of early first attendance were identified.

### Use of anti-malarials during pregnancy

Eighty percent of women had heard of intermittent preventive treatment (IPTp) with sulphadoxine-pyrimethamine (SP) to prevent malaria in pregnant women and 55% of respondents reported receiving at least one dose of IPTp. Table 2 presents the data on use of IPTp by facility, and also categorizes the respondents by gestation age, to account for their different exposure times. The outcome of having received one dose of IPTp (among those ≥5 months gestation) or two doses of IPTp (among those ≥7 months gestation) was associated in bivariate analysis only with the antenatal facility attended (chi-squared test for association; P = 0.001), and not with any other individual characteristics, including age, marital status, socio-economic status, education level, attendance at health education or timing of first ANC visit.

**Table 2:** Use of anti-malarial drugs during pregnancy

**District**	**Pangani**		**Tanga**		**Korogwe**		**TOTAL**
**Facility level**	**District Hospital**	**Dispensary**	**District Hospital**	**Health Centre**	**District Hospital**	**Dispensary**	

**Respondents per facility**	17	13	32	28	19	10	**119**
**Any anti-malarials taken during this pregnancy**							
**As treatment, n (%)**	3 (17.7)	2 (15.4)	8 (25.0)	5 (17.9)	4 (21.1)	1 (10.0)	**23 (19.3)**
**As prevention, n (%)**	12 (70.6)	9 (69.2)	12 (37.5)	14 (50.0)	11 (57.9)	9 (90.0)	**67 (56.3)**
**Heard of IPTp, n (%)**	14 (82.4)	11 (84.6)	19 (59.4)	24 (85.7)	18 (94.7)	10 (100)	**96 (80.7)**
**Received any doses IPTp**^†^							
**Overall, n (%)**	9 (52.9)	7 (53.9)	11 (34.4)	15 (53.6)	14 (73.7)	9 (90.0%)	**65 (54.6)**
Uptake among those ≥ 5 months gestation*	64.3%	77.8%	40.0%	62.5%	100%	100%	**67.0%**
**Received ≥ 2 doses IPTp**^†^							
**Overall, n (%)**	7 (41.2)	5 (38.5)	4 (12.5)	5 (17.9)	6 (31.6)	4 (40.0)	**31 (26.1)**
Uptake among those ≥ 7 months gestation**	87.5%	75.0%	16.7%	30.8%	85.7%	66.7%	**48.2%**

^†^Respondents who had not heard of IPTp were not asked about doses received

*Denominator is number of respondents who were 5 months gestation or later

**Denominator is number of respondents who were 7 months gestation or later

### Timing of IPTp delivery

**Figure 1:**
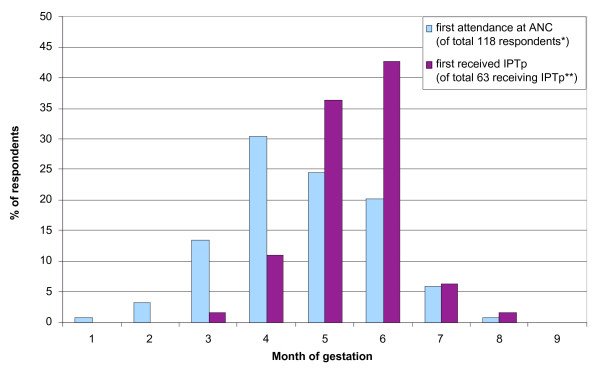
**Comparison of timing of first attendance at antenatal clinic with timing of first dose of IPTp**. *Information missing for one respondent. **Information missing for two respondents.

The timing of first IPTp dose within the study population is illustrated in Figure 1 and demonstrates that when IPTp was given, it was generally first given during the fifth or sixth month of gestation. This is consistent with the national guidelines for delivery of IPTp, which recommends giving the first dose between 20 and 24 weeks gestation and the second dose between 28 and 32 weeks gestation [10]. According to the respondents' reports, 13% (8/63) of first IPTp doses were given earlier than the scheduled timing of five months gestation, however Figure 1 also shows that almost half of the total respondents attended ANC prior to five months gestation, and thus the large majority of those early attendees (49/57; 86%) were not given IPTp on that first visit.

### Barriers to the delivery of IPTp

For those who did not receive IPTp as scheduled, the key reason reported was that it was not offered by ANC staff. Only one woman said she had refused SP when given, due to perceived negative physical effects.

Of the 12 ANC staff interviewed, all said they knew the schedule for delivery of IPTp and 8/12 (67%) could describe it correctly (3/12, 25% partly correctly; 1/12, 8% incorrectly). The only two reports of deviation from the IPTp schedule were in giving an additional SP dose outside the recommended time windows, however two staff at one clinic also reported that SP was not currently stocked in their clinic for use in IPTp.

The observations of consultations were limited by the fact that women of different gestation ages and number of prior visits received different information and interventions, however they did highlight the variability between facilities in the degree to which IPTp was discussed and delivered, and specifically that women at four months or less gestation were commonly told that they should come back at five months to receive the first dose of IPTp, as was reported also by the women themselves in interview. Data from observations of consultations also confirmed that at the facility in which IPTp coverage was lowest, staff were currently not dispensing SP at all. At this clinic, patients were told that SP was out of stock and that they would need to purchase it elsewhere, however the cross-sectional nature of this study meant that it was not possible to determine whether these women did obtain SP from another source, nor how long SP remained out of stock at this clinic.

## Discussion

The results of this study suggest that the timely uptake of malaria preventive interventions by pregnant women in this region of north-east Tanzania depends more on the practices of health workers at the antenatal clinic (ANC) attended, rather than the individual characteristics of the pregnant woman or the timing of her first attendance at ANC.

### Early ANC attendance – a missed opportunity for timely delivery of IPTp

The vast majority of respondents attended ANC at least once before the third trimester of pregnancy, and approximately half made their first visit early, ie. during or before the fourth month of gestation, which is consistent with other reports of ANC attendance in Tanzania [11]. However, no evidence was found of an association between early ANC attendance and uptake of intermittent preventive treatment (IPTp), a key malaria preventive strategy. This suggests that efforts to encourage timely ANC attendance alone are unlikely to improve the uptake of this intervention, at least where other barriers to delivery exist.

Although the coverage of IPTp was variable among the study sites, where it was given by antenatal clinics the timing of delivery was generally in accordance with the national guidelines [10]. Knowledge of the recommended schedule for delivery of IPTp was high among clinic staff, and the guidelines were well-publicized with several clinics displaying posters and most providing pamphlets describing the appropriate gestation ages for first and second doses. Although this demonstrates the effectiveness of clear and simple guidelines in achieving high compliance, the narrow gestational range specified by the schedule limits the potential benefit of IPTp early in pregnancy. A large proportion of women in this study who attended ANC at four months gestation or earlier were required to wait until a later visit in order to receive IPTp, delaying the benefits of treatment and risking the possibility that they do not return for a second antenatal visit. This represents a missed opportunity for early delivery of this important intervention, and suggests that encouragement of earlier ANC attendance could have only a limited effect on the uptake of IPTp. Recent evidence in HIV-positive women in Zambia suggests that two or more doses of IPTp delivered between 16 and 36 weeks gestation is associated with improved maternal and foetal outcomes compared with a single dose, but with a threshold effect of little marginal benefit above two doses [22]. Whether this principle extends to non-HIV infected women and to delivery of IPTp in the early weeks of the second trimester is not known but seems highly plausible, and these results highlight the importance of maximising the proportion of women accessing the recommended two doses of IPTp.

The national policy in Tanzania for first-line treatment of malaria has changed to the artemisinin-based combination drug artemether-lumefantrine, in the face of growing resistance to the conventional anti-malarials chloroquine and SP [10]. The guidelines for use of SP for IPTp remain unchanged however, as the efficacy requirement for presumptive treatment is not as high as for treatment of clinical malaria [10] and the safety of artemether-lumefantrine in pregnancy is not yet established [23]. The present study identified potential confusion at the clinic level around this policy change. In one facility SP was not stocked and of the women interviewed in this clinic, one reported that she had been told that the hospital had stopped its distribution of SP for the moment, and another that SP had 'expired' and was no longer functioning. Although the staff interviewed at this clinic confirmed that SP was out of stock, the interview questionnaire did not permit further investigation of the reasons for this or the perceived implications of the policy change on the use of SP for IPTp. The lack of SP at this clinic presented a barrier to the timely delivery of IPTp, and the indication that this may be due to confusion over the change in malaria treatment policy highlights the need for training and dissemination of information to the periphery as part of the implementation of the new policy.

The data on the uptake of IPTp reported here is limited in its generalizability beyond the study population, due to the small sample size of both facilities and individual respondents. Although clinics were selected to represent a range of settings and levels of health facility, sampling was not done randomly and therefore may be a source of bias. However, the national guidelines determining the timing of delivery of IPTp apply throughout Tanzania, and although the timing of first attendance at antenatal care may vary within other populations, the pattern observed in this study was consistent with other reports of ANC attendance in Tanzania [11]. This suggests that the results presented here may apply elsewhere in Tanzania, at least where there is early ANC attendance, and justify a larger survey to further investigate the issues of timing and external constraints to delivery of IPTp.

### Potential benefits and risks of earlier delivery of IPTp

There is currently little known about the effect of malaria in early pregnancy on pregnancy outcomes [24]. However, the prevalence and severity of malaria infection is known to be highest during early pregnancy [16] and a recent review of the pathogenesis of malaria-associated foetal growth restriction hypothesized a mechanism by which malaria infection early in pregnancy may impair placental blood flow, leading to poor pregnancy outcomes [25]. Some evidence of an effect of malaria infection during the first trimester on low birthweight has also been reported by others [17], and together this evidence suggests that the maximum benefit of IPTp may be gained by delivery of the first dose early in pregnancy. This potential benefit needs to be weighed against any potential risks associated with delivery of SP early in pregnancy, given theoretical concerns over the association of folate antagonists with neural tube defects and other congenital abnormalities [26,27]. However, SP has a very good safety profile in pregnancy with extensive clinical trial data supporting its safety when delivered in the second and third trimesters [28], and is considered by the WHO to be one of the anti-malarial drugs that is safe to use for treatment during the first trimester of pregnancy [23]. The considerable evidence for the safety of SP during the second trimester of pregnancy, and the observation in this study that up to 75% of women may first attend antenatal clinic during the second trimester, support the conclusion that it may be operationally possible, safe and of potential benefit to maternal health to offer the first dose of IPTp earlier in the second trimester than is currently prescribed by Tanzanian national guidelines.

While SP continues currently to be recommended for use in IPTp, growing drug resistance especially in East Africa means that an alternative is likely to be required in the near future. The efficacy and safety of many candidate anti-malarials has not yet been demonstrated in pregnant women [29], and there is some indication from animal models that artemisinin compounds may be associated with early pregnancy loss, although this has not been observed in the limited human data [29]. Further research is needed both to establish the effect of malaria infection at different gestational stages on pregnancy outcomes, and to examine the safety profile of intermittent delivery of SP and other candidate anti-malarials during the first half of pregnancy, thus providing evidence to inform the timing of delivery of preventive interventions.

## Conclusion

Where IPTp has been adopted as part of a national policy for malaria control, ongoing research is needed into operational barriers to its delivery. These barriers may be common to many implementation settings within and between countries. Given the observation in this study of generally good adherence by clinic staff to the national IPTp dosing schedule, a change in policy in Tanzania to recommend earlier delivery of the first dose of SP (for example at 4 months gestation) may improve IPTp coverage in areas where a large proportion of women attend ANC early in pregnancy. At the same time, local variations in availability of SP at antenatal clinics need to be recognized and addressed, in particular to provide accurate information about current guidelines for SP use as IPTp, in the context of increasing SP resistance and the change in policy for first-line malaria treatment.

## Authors' contributions

KA was responsible for the study design, development of protocols, training of field staff, data collection and analysis, and preparation of the manuscript. HR oversaw the study design and data collection, facilitated the recruitment of study sites and provided guidance on the data analysis and development of the manuscript. TM assisted in the development of the survey instruments, and provided guidance on the study design, data analysis and the development of the manuscript. PC co-ordinated the recruitment of participants at the study sites and the training of field staff, and assisted with development and translation of survey instruments. PM had input into the study design and facilitated the recruitment of study sites. All authors read and approved the final manuscript.

## References

[B1] Guyatt HL (2001). The epidemiology and burden of Plasmodium falciparum-related anaemia among pregnant women in sub-Saharan Africa. Am J Trop Med Hyg.

[B2] Guyatt HL, Snow RW (2004). Impact of malaria during pregnancy on low birth weight in sub-Saharan Africa. Clin Microbiol Rev.

[B3] Steketee RW, Nahlen BL, Parise ME, Menendez C (2001). The burden of malaria in pregnancy in malaria-endemic areas. Am J Trop Med Hyg.

[B4] World Health Organization (2004). A strategic framework for malaria prevention and control during pregnancy in the African region.

[B5] Schultz LJ, Steketee RW, Chitsulo L, Macheso A, Kazembe P, Wirima JJ (1996). Evaluation of maternal practices, efficacy, and cost-effectiveness of alternative antimalarial regimens for use in pregnancy: chloroquine and sulfadoxine-pyrimethamine. Am J Trop Med Hyg.

[B6] Shulman CE, Dorman EK, Cutts F, Kawuondo K, Bulmer JN, Peshu N, Marsh K (1999). Intermittent sulphadoxine-pyrimethamine to prevent severe anaemia secondary to malaria in pregnancy: a randomised placebo-controlled trial. Lancet.

[B7] van Eijk AM, Ayisi JG, ter Kuile FO, Otieno JA, Misore AO, Odondi JO, Rosen DH, Kager PA, Steketee RW, Nahlen BL (2004). Effectiveness of intermittent preventive treatment with sulphadoxine-pyrimethamine for control of malaria in pregnancy in western Kenya: a hospital-based study. Trop Med Int Health.

[B8] Crawley J, Hill J, Yartey J, Robalo M, Serufilira A, Ba-Nguz A, Roman E, Palmer A, Asamoa K, Steketee R (2007). From evidence to action? Challenges to policy change and programme delivery for malaria in pregnancy. Lancet Infect Dis.

[B9] Roll Back Malaria (2004). Tanzania RBM Country Consultative Mission Final Report.

[B10] Ministry of Health (2006). National Guidelines for Malaria Diagnosis and Treatment. Malaria Control Series (No 11).

[B11] Hanson K, Marchant T, Mponda H, Nathan R, Bruce J (2006). Monitoring and evaluation of the Tanzanian National Voucher Scheme: Report on 2006 TNVS Household, Facility services and Facility users surveys (a comparison between baseline and 12 month follow-up).

[B12] Hill J, Kazembe P (2006). Reaching the Abuja target for intermittent preventive treatment of malaria in pregnancy in African women: a review of progress and operational challenges. Trop Med Int Health.

[B13] Mubyazi G, Bloch P, Kamugisha M, Kitua A, Ijumba J (2005). Intermittent preventive treatment of malaria during pregnancy: a qualitative study of knowledge, attitudes and practices of district health managers, antenatal care staff and pregnant women in Korogwe District, North-Eastern Tanzania. Malar J.

[B14] Holtz TH, Kachur SP, Roberts JM, Marum LH, Mkandala C, Chizani N, Macheso A, Parise ME (2004). Use of antenatal care services and intermittent preventive treatment for malaria among pregnant women in Blantyre District, Malawi. Trop Med Int Health.

[B15] Nganda RY, Drakeley C, Reyburn H, Marchant T (2004). Knowledge of malaria influences the use of insecticide treated nets but not intermittent presumptive treatment by pregnant women in Tanzania. Malar J.

[B16] Brabin BJ (1983). An analysis of malaria in pregnancy in Africa. Bull World Health Organ.

[B17] Cottrell G, Mary JY, Barro D, Cot M (2007). The importance of the period of malarial infection during pregnancy on birth weight in tropical Africa. Am J Trop Med Hyg.

[B18] Drakeley CJ, Carneiro I, Reyburn H, Malima R, Lusingu JP, Cox J, Theander TG, Nkya WM, Lemnge MM, Riley EM (2005). Altitude-dependent and -independent variations in Plasmodium falciparum prevalence in northeastern Tanzania. J Infect Dis.

[B19] Maxwell CA, Chambo W, Mwaimu M, Magogo F, Carneiro IA, Curtis CF (2003). Variation of malaria transmission and morbidity with altitude in Tanzania and with introduction of alphacypermethrin treated nets. Malar J.

[B20] Drakeley CJ, Corran PH, Coleman PG, Tongren JE, McDonald SL, Carneiro I, Malima R, Lusingu J, Manjurano A, Nkya WM, Lemnge MM, Cox J, Reyburn H, Riley EM (2005). Estimating medium- and long-term trends in malaria transmission by using serological markers of malaria exposure. Proc Natl Acad Sci U S A.

[B21] Maxwell CA, Rwegoshora RT, Magesa SM, Curtis CF (2006). Comparison of coverage with insecticide-treated nets in a Tanzanian town and villages where nets and insecticide are either marketed or provided free of charge. Malar J.

[B22] Gill CJ, Macleod WB, Mwanakasale V, Chalwe V, Mwananyanda L, Champo D, Mukwamataba D, Chilengi R, Thea DM, Hamer DH (2007). Inferiority of Single-Dose Sulfadoxine-Pyrimethamine Intermittent Preventive Therapy for Malaria during Pregnancy among HIV-Positive Zambian Women. J Infect Dis.

[B23] World Health Organization (2006). Guidelines for the treatment of malaria.

[B24] Desai M, ter Kuile FO, Nosten F, McGready R, Asamoa K, Brabin B, Newman RD (2007). Epidemiology and burden of malaria in pregnancy. Lancet Infect Dis.

[B25] Rogerson SJ, Boeuf P (2007). New approaches to pathogenesis of malaria in pregnancy. Parasitology.

[B26] Hernandez-Diaz S, Werler MM, Walker AM, Mitchell AA (2000). Folic acid antagonists during pregnancy and the risk of birth defects. N Engl J Med.

[B27] Hernandez-Diaz S, Werler MM, Walker AM, Mitchell AA (2001). Neural tube defects in relation to use of folic acid antagonists during pregnancy. Am J Epidemiol.

[B28] Peters PJ, Thigpen MC, Parise ME, Newman RD (2007). Safety and toxicity of sulfadoxine/pyrimethamine: implications for malaria prevention in pregnancy using intermittent preventive treatment. Drug Saf.

[B29] Vallely A, Vallely L, Changalucha J, Greenwood B, Chandramohan D (2007). Intermittent preventive treatment for malaria in pregnancy in Africa: what's new, what's needed?. Malar J.

